# Associations between smoking trajectories, smoke-free laws and cigarette taxes in a longitudinal sample of youth and young adults

**DOI:** 10.1371/journal.pone.0246321

**Published:** 2021-02-11

**Authors:** Dorie E. Apollonio, Lauren M. Dutra, Stanton A. Glantz

**Affiliations:** 1 Department of Clinical Pharmacy, School of Pharmacy, University of California San Francisco, San Francisco, California, United States of America; 2 Center for Health Analytics, Media, and Policy, RTI International, Berkeley, California, United States of America; 3 Center for Tobacco Control Research and Education, University of California San Francisco, San Francisco, California, United States of America; University of California San Diego School of Medicine, UNITED STATES

## Abstract

Cigarette smoking patterns vary within the population, with some individuals remaining never smokers, some remaining occasional users, and others progressing to daily use or quitting. There is little research on how population-level tobacco control policy interventions affect individuals within different smoking trajectories. We identified associations between tobacco control policy interventions and changes across different smoking trajectories among adolescents and young adults. Using 15 annual waves of data drawn from the National Longitudinal Survey of Youth 1997 (NLSY97), we applied a group-based trajectory model to identify associations between days smoked per month, comprehensive smoke-free laws, cigarette tax rates, and known socio-demographic risk factors for membership in different smoking trajectories. Comprehensive smoke-free laws were associated with reduced risk of initiation and reductions in days smoked per month for all trajectories other than occasional users. Higher tax rates were associated with reduced risk of initiation and days smoked for all trajectories other than established users. Overall, population-based tobacco control policies, particularly comprehensive smoke-free laws, were associated with reduced smoking. Tobacco taxes primarily reduced risk of initiation and use among never smokers, experimenters, and quitters, consistent with previous research suggesting that tobacco manufacturers lower prices after tax increases to reduce the cost of continued smoking for established users. These results provide support for expanding smoke-free laws and establishing a minimum tobacco floor price, which could improve public health by reducing the risk of initiation as well as use among occasional and established smokers.

## Introduction

Tobacco use is the leading preventable cause of death in the US, killing over 480,000 people each year [1]. Most of these deaths occur among cigarette smokers, 80% of whom begin smoking by age 18 [2] and 99% of whom begin by age 25 [3]. The transition from experimentation to established smoking generally occurs in the late teens and early 20s [4, 5].

Tobacco use patterns vary within the population, with some people never smoking, some remaining occasional users, and others progressing to daily use or quitting. Existing research has identified 4–6 trajectories of smoking, typically classified as never-smokers, experimenters/occasional users, reducers or quitters, those who start smoking young and quickly become daily smokers, and those who start smoking as young adults and become daily smokers [6–12]. Studies of smoking trajectories have primarily focused on identifying risk factors at the individual or family level [7, 8, 10–17], determining associations between trajectory type and health outcomes [6, 9, 18], and assessing links between trajectories and use of other products [19]. There has been little attention to how tobacco control policies affect these trajectories. Understanding the factors that influence smoking initiation and the transition to regular smoking is critical to developing tobacco control interventions that improve public health [8]. The identification of distinct trajectories of smoking behavior, which arise from different determinants, offers a more accurate way of describing and understanding behavior and makes it possible to design interventions for specific groups instead of assuming that interventions have comparable effects across the entire population. This is similar to the market segmentation that the tobacco industry employs in designing its products and marketing strategies [20, 21]. Tobacco control policies may reduce smoking at the population level by reducing the risk of initiation, encouraging users to quit once they have begun smoking, discouraging ex-smokers from relapsing, or some combination of these effects [22, 23].

Population-based studies have found that in addition to protecting people from secondhand smoke, smoke-free laws stimulate quit attempts, support smoking cessation, and contribute to reducing cigarette smoking among adolescents and young adults [24, 25]. One study used 11 years of data from the National Longitudinal Survey of Youth 1997 (NLSY97) to assess the effects of smoking restrictions on respondents’ smoking behavior and found laws for smoke-free workplaces, but not bars, were associated with reduced smoking initiation; smoke-free bar laws were associated with lower odds of current smoking and fewer days of smoking among current smokers [26]. However, literature on the effects of smoke-free laws on smoking trajectories is limited, reflecting in part the difficulty of collecting longitudinal data on tobacco use and policies (which are complicated by substantial differences in smoke-free laws between localities and states), as well as the issue that many growth models do not accommodate time-varying covariates.

Tax increases reduce smoking and increase quit attempts at the population level [27, 28], but research seeking to assess their effects on established smokers has identified inconsistent effects [29–32]. Popular media reports on tax increases typically assume that established smokers will not quit and instead will switch to discount brands and seek out lower-taxed or illicit tobacco [33, 34]. However youth and young adults who are still forming smoking habits are highly price-sensitive [35], suggesting that tax increases could reduce initiation or discourage the transition to established smoking; higher prices could make starting smoking, or continuing to smoke, too costly. A previous study based on 11 years of NLSY97 data found that taxes were associated with a lower risk of initiation into smoking but did not affect current smoking [26], but this study treated all respondents as a single group and did not account for smoking trajectories. A cross-sectional latent class analysis in Minnesota found that a cigarette tax increase was associated with less smoking across classes [27]. Despite literature suggesting that taxes deter smoking, multiple studies have found that daily smokers use price minimization strategies, such as discount brands, coupons, and purchasing in lower tax jurisdictions, to offset increases in excise taxes [36–41]. The tobacco industry has also lobbied for changes in tax calculations and redesigned marketing to focus on discounts and coupons to undercut the effects of tobacco tax increases [42, 43]. In response, policies have attempted to establish counter-measures such as tobacco minimum floor prices [40, 42, 44].

Little research exists on the role that changes in taxes over time play in the transition from adolescent smoking initiation to established use [7, 27]. Even less exists on associations between policies and smoking behavior within and across smoking trajectories. While one study considered the role of media campaigns on smoking behavior over time, we were unable to identify any previous trajectory-based research assessing smoke-free laws [24, 45]. Examining the influence of policies on smoking trajectories has the potential to identify heterogeneous effects of existing policies and the need for tailored smoking prevention and cessation approaches.

Our current study addressed this gap by assessing the effects of tobacco control policies known to be effective at the population level on trajectories of use for adolescents as they become young adults. We anticipated that smoke-free laws would reduce the risk of tobacco initiation and use across all smoking trajectories and that tax increases would reduce the risk of initiation among never smokers but not reduce use among current established smokers. The authors previously published research that identified trajectories of smoking using NLSY97 data; this earlier paper did not consider the impact of the policy environment (smoke-free laws and taxes) [8]. Adding these variables, which changed over time, is the important new contribution of this paper. This new analysis provides guidance for policy makers seeking to reduce tobacco initiation and use across the population and reduce relapse to use among those who have quit.

## Methods

Using longitudinal data collected over 15 years, we identified associations between smoking patterns, local smoke-free laws, and tobacco tax rates for young people in multiple smoking trajectories while controlling for known individual risk factors.

We used weighted data from the NLSY97, a nationally representative sample of individuals born between 1980 and 1984 [46]. The NLSY97 dataset is collected by the US Bureau of Labor Statistics (BLS). The initial sample was drawn using the National Opinion Research Center’s (NORC) 1990 master probability US sample. Data collection began in 1997 when participants were 12 to 16 years old and continued with annual follow-ups through 2011 (data collection thereafter was biennial). Surveys were completed using computer-assisted in-person and telephone interviews. Flowcharts for participants’ progress through the survey instruments in each year of data collection are provided at the U.S. Bureau of Labor Statistics National Longitudinal Surveys website [47]. We extracted variables from the NLSY97 at the NLS Investigator site by selecting variables describing participant attitudes, behavior, and demographics (detailed below), as well as sampling weights, from the complete list provided by NLSY97 and downloading them for analysis. The public use variables included in this analysis can be obtained through NLS Investigator (https://www.nlsinfo.org/investigator/pages/login) and geographical data by making a request to BLS for access to data restricted under the Confidential Information Protection and Statistical Efficiency Act (CIPSEA) (https://www.bls.gov/rda/home.htm). The NLSY97 panel began with 8,984 participants, and by the 15^th^ wave in 2011, had experienced 17.4% attrition (n = 7,423). We used participant geocodes (states and counties) from the BLS restricted-use dataset to link smoke-free law coverage and tobacco taxes to participants’ survey data.

### Outcome variables

Our primary outcome measure was days smoked per month. There is no widely accepted measure of smoking for trajectory models addressing smoking among youth and young adults. Previous studies have compared four different measures of smoking: mean cigarettes smoked per day, cigarettes per day on days smoked, days smoked per month, and total cigarettes per month; of these, days smoked per month provided maximum differentiation between trajectories, captured smoking progression over time, uniquely described smoking behavior, and avoided problems of instability over time created by the use of measures such as cigarettes per day or month [8, 48].

### Predictor variables

We included measures of policy interventions and measures of socio-demographic characteristics that have been identified as predictors of trajectory membership in multiple previous studies [7, 8, 10–17].

#### Policy intervention variables

We included two types of policy interventions as time-varying covariates, measured annually: smoke-free laws and tax rates. NLSY97 geocodes provide Federal Information Processing Standards (FIPS) codes that identify the county of each respondent, which made it possible for us to match policy variables with survey responses.

*Smoke-free laws*. The smoke-free law variable accounted for both state and local (place-based, i.e., counties, cities, and towns) laws because state-level smoke-free laws are often weaker than local ordinances [49]. RTI International, which has collected information on smoke-free laws by locality throughout the US since the 1990s, provided data on state and local smoke-free law coverage. RTI’s database is quarterly and calculates smoke-free law coverage by combining smoke-free law data from the American Nonsmokers’ Rights Foundation database [50] with annual population data from the U.S. Census Bureau [51–56]. The database was created by entering smoke-free laws individually by effective date (the date in which all components of the law were in effect) and using statistical code to estimate the fraction of the population covered by smoke-free laws at the state, county, and place level for workplaces, restaurants, and bars. This figure can also be interpreted as the probability that a given individual in a given locality will be covered by smoke-free laws. The database only includes 100% smoke-free laws, which are defined by the American Nonsmokers’ Rights Foundation as smoke-free laws with few or no exceptions (i.e., loopholes) [57].

This study used 100% smoke-free law coverage by workplace, restaurant, and bar laws as our measure, consistent with US Centers for Disease Control and Prevention (CDC) guidelines [58]. When 100% of the county’s population was covered by smoke-free laws (because of county and/or state law), the county was assigned a 1. For counties not covered by any state, county, or local smoke-free law, the county was assigned a 0. For counties with no state or county laws but with local laws, the county was assigned a quantity between 0 and 1 that represented the proportion of the county’s population covered by smoke-free laws (calculated by linking the laws to US Census Bureau data for population estimates).

We lagged smoke-free coverage by a year (an established method in tobacco and cannabis research [26, 59]) to ensure smoke-free laws had been fully implemented and had time to affect behavior before each year of NLSY97 data collection.

*Tobacco taxes*. Taxes are a more accurate measure of tobacco control policy than prices [60]. Tax data were drawn from *Tax Burden on Tobacco* [61], an annually updated resource archived by the US Centers for Disease Control and Prevention (CDC) that lists statewide tobacco tax rates in every year since 1970. Nominal taxes were converted to 2011 dollars using average Consumer Price Index data for all goods and services. We did not include local tax rates; very few US localities can impose tobacco excise taxes [62, 63]. Changes in taxes were assumed to take effect the same year, and the amount of the tax in each year, in dollars, was natural log-transformed due to skew.

#### Socio-demographic variables

Socio-demographic variables, most of which were assessed during the first year of data collection (1997; except gender, which was assessed in 2011) included: gender (female (reference)/male), race/ethnicity (non-Hispanic White (reference), non-Hispanic Black, Hispanic, or non-Hispanic mixed/other, biological mother’s education as a measure of socioeconomic status (SES; less than General Education Diploma (GED)/high school diploma (reference), GED/high school graduate, Associate Degree (AA), Bachelor of Arts (BA) or Bachelor of Science (BS) degree, or graduate/professional degree), and employment/school enrollment at age 16 as a measure of engagement (enrolled in school but not employed (reference), employed but not enrolled, enrolled and employed, or neither employed nor enrolled). Additional categorical variables included non-two parent family (any respondent not living with both biological parents), ever use of alcohol, and ever use of cannabis (measured at baseline given potential collinearity with policy changes such as smoke-free laws). To assess young adult characteristics, we included two categorical variables, ever married and having one or more children, measured at age 26 given their low probability or illegality at baseline. Household income was coded on a 4-point scale relative to the previous year’s federal poverty line into four quartiles from below poverty line (0), up to 199%, 200–299%, and 300%+ (3).

We included age at baseline to assess cohort effects. As a measure of mental health status, depression (asked in 2000) was coded using mean score for the five-question adapted NLSY97 Mental Health Inventory, with higher values reflecting higher levels of depression. All questions were coded on a four-point response scale ranging from “none of the time” (0) to “all of the time” (3), and included “How much of the time during the last month have you…” “been a nervous person,” “felt calm and peaceful,” “felt down or blue,” “been a happy person,” and “felt so down in the dumps that nothing could cheer you up.” Peer smoking at baseline was coded on a five-point response scale based on the question “What percentage of the kids in your grade [when you were last in school] smoke[d] cigarettes?” and coded from “almost none (less than 10%)” (0) to “almost all (more than 90%)” (4). We also included responses to the 2008 question “When I was in school, I used to break rules quite regularly” coded on a 7-point response scale from disagree strongly (0) to agree strongly (6).

#### Analytical strategy

We created a group-based trajectory model [64] (also known as a growth mixture model) using the Stata version 15 “traj” plugin (based on PROC TRAJ developed for SAS) with a zero-inflated Poisson model due to the large numbers of zeroes in the data (never smokers) [65–67]. Within each trajectory, smoking was modeled as a function of time. The group-based trajectory model clumps individual trajectories into distinctive clusters to permit identification of the characteristics of individuals in these clusters [67]. This model allows investigation of differences across groups within a population and assessment of patterns of shifting behavior over time using maximum likelihood methods to estimate the parameters of the model (as opposed to the multivariate continuous distribution functions used by hierarchical and latent class methodologies) [67, 68]. The group-based trajectory model requires making a preliminary decision about the number of assumed trajectories. We specified five trajectories based on a previous latent class growth analysis of the same years of the NLSY97 data (1997 to 2011) that modeled days smoked in the past 30 days [8].

We used traj’s built-in capacity to calculate the effect of time-varying covariates, in this case, policies (assessed annually), on the probability of membership in each trajectory [67]. Traj conducts these calculations by generalizing the specification of the polynomial function of time, which defines the shape of the trajectory, to include covariates. We also used traj’s built-in ability to calculate the effect of time-invariant covariates, in this case, the socio-demographic risk factors [7, 8, 10–17], on the trajectory itself. Traj uses a generalized logistic function for these calculations. We assessed robustness of the final model by verifying that the directionality of covariates remained the same across all steps of stepwise deletion of risk factor variables and when policy variables were log-transformed and/or lagged [69].

Traj uses listwise deletion for missing data. Missing data led to the exclusion of 43.5% of the 2011 sample. Approximately half of these exclusions represented individuals for which NLSY97 did not report a geocode, and the remainder were for participants with incomplete risk factor data.

We used the coefficients in the model associated with the effect of proportion of the population covered by 100% smoke-free workplace, restaurant, and bar laws, b_S_, and the coefficient associated with the effect of an increase of one natural log unit in tobacco tax, b_T_, to compute the tax equivalent in 2011 dollars of a smoke-free law by solving b_S_ = b_T_ ln(T) for T; T = exp(b_S_/b_T_).

## Results

**Fig 1:**
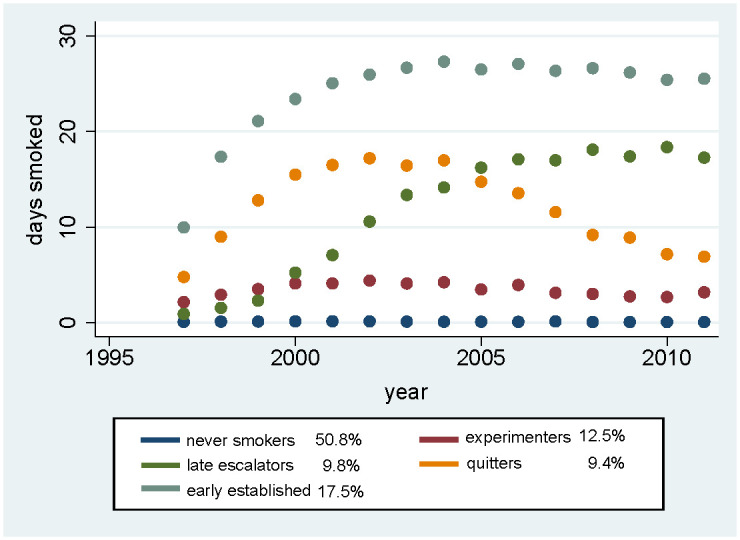
Five identified trajectories of smoking behavior in the National Longitudinal Survey of Youth (NLSY97).

The group-based trajectory analysis identified trajectories (Fig 1) consistent with the results of the previous work [8]. The first trajectory consisted of never smokers (50.8%). Given that we identified similar trajectories as in past research, we used the same naming convention for the other four trajectories: experimenters (12.5%), late escalators (9.8%) quitters (9.4%), and early established smokers (17.5%). A classification as “experimenter” was associated with less than 10 smoking days per month in every year of data collection. “Quitters’” consumption peaked in early adulthood, between ages 18–22 years, and then declined. “Late escalators” peaked with respect to days smoked per month at ages 22–26 years, in contrast to “early escalators,” who did so at ages 19–23 years.

Regarding the effects of policies on the trajectories (i.e., days of smoking within each trajectory), as the probability of being covered by comprehensive smoke-free law increased, predicted days of use in a month decreased in all trajectories other than experimenters, where coverage by comprehensive smoke-free laws was associated with more days of smoking (Table 1). The effect was most substantial for quitters (-1.99±0.04 [SE] days/month) and never smokers (-0.36±0.10 days/month) and was also associated with reduced days of smoking in a month for late escalators (-0.13±0.01 days/month) and early established smokers (-0.05±0.01 days/month). Experimenters (+0.81±0.03 days/month) were an exception.

**Table 1:** Policies and risk factors associated with smoking in different trajectories (n = 4,192; significant associations in bold).

	Never smokers		Experimenters		Late escalators		Quitters		Early established	
**Time-varying** (coefficient±SE)										
Tax rate	**-0.15±0.02**	p<0.001	**-0.32±0.01**	p<0.001	**0.83±0.01**	p<0.001	**-0.02±0.01**	p<0.001	**0.08±0.002**	p<0.001
Comprehensive smoke-free ordinance	**-0.36±0.10**	p<0.001	**0.81±0.03**	p<0.001	**-0.13±0.01**	p<0.001	**-1.99±0.04**	p<0.001	**-0.05±0.01**	p<0.001
**Risk factors** (OR (95% CI))										
Gender										
• Female	baseline		reference		reference		reference		reference	
• Male	baseline		**1.37 (1.11–1.70)**	p = 0.004	**1.78 (1.40–2.26)**	p<0.001	**1.56 (1.22–2.00)**	p<0.001	**1.27 (1.02–1.58)**	p = 0.030
Race/ethnicity										
• Non-Hispanic White	baseline		reference		reference		reference		reference	
• Non-Hispanic Black	baseline		0.76 (0.57–1.01)	p = 0.062	0.75 (0.56–1.01)	p = 0.062	**0.48 (0.35–0.66)**	p<0.001	**0.20 (0.15–0.26)**	p<0.001
• Hispanic	baseline		1.19 (0.90–1.56)	p = 0.226	**0.62 (0.44–0.87)**	p = 0.006	**0.49 (0.34–0.69)**	p<0.001	**0.18 (0.13–0.25)**	p<0.001
• Non-Hispanic mixed/other	baseline		0.78 (0.40–1.52)	p = -.469	1.69 (0.97–2.95)	p = 0.064	0.49 (0.21–1.18)	p = 0.113	0.68 (0.37–1.26)	p = 0.222
Mother’s highest education										
• Less than GED/HS diploma	baseline		reference		reference		reference		reference	
• GED/HS graduate	baseline		0.97 (0.71–1.32)	p = 0.856	0.95 (0.68–1.32)	p = 0.763	1.17 (0.81–1.70)	p = 0.400	1.34 (0.97–1.86)	p = 0.076
• AA/BA/BS	baseline		1.10 (0.77–1.57)	p = 0.614	0.86 (0.58–1.28)	p = 0.462	1.12 (0.73–1.72)	p = 0.590	1.24 (0.85–1.80)	p = 0.257
• Graduate/professional degree	baseline		0.90 (0.54–1.51)	p = 0.689	0.82 (0.46–1.46)	p = 0.499	0.57 (0.28–1.15)	p = 0.118	0.72 (0.40–1.28)	p = 0.262
Employment/school enrollment status										
• In school, not employed	baseline		reference		reference		reference		reference	
• Employed, not in school	baseline		1.11 (0.66–1.87)	p = 0.685	1.09 (0.63–1.89)	p = 0.748	**1.82 (1.08–3.06)**	p = 0.024	**3.07 (2.02–4.66)**	p<0.001
• Employed and in school	baseline		0.98 (0.78–1.22)	p = 0.834	**0.78 (0.61–0.99)**	p = 0.043	1.06 (0.82–1.39)	p = 0.649	0.86 (0.68–1.09)	p = 0.214
• Neither in school nor employed	baseline		1.25 (0.74–2.11)	p = 0.407	1.47 (0.89–2.43)	p = 0.134	**2.30 (1.36–3.90)**	p = 0.002	**3.91 (2.55–5.98)**	p<0.001
Non-two parent family	baseline		0.96 (0.77–1.20)	p = 0.733	1.04 (0.82–1.33)	p = 0.731	**1.41 (1.09–1.81)**	p = 0.008	**1.66 (1.33–2.06)**	p<0.001
Ever drank alcohol	baseline		**1.69 (1.35–2.13)**	p<0.001	**1.48 (1.15–1.92)**	p = 0.003	**1.82 (1.39–2.38)**	p<0.001	**1.91 (1.51–2.41)**	p<0.001
Even used cannabis	baseline		**1.84 (1.36–2.49)**	p<0.001	**1.67 (1.16–2.39)**	p = 0.005	**2.86 (2.10–3.90)**	p<0.001	**3.56 (2.71–4.66)**	p<0.001
Household income	baseline		1.05 (0.93–1.20)	p = 0.424	0.89 (0.77–1.03)	p = 0.105	1.05 (0.90–1.22)	p = 0.517	0.92 (0.81–1.04)	p = 0.194
Age (in 1997)	baseline		**0.87 (0.80–0.94)**	p<0.001	**0.77 (0.70–0.84)**	p<0.001	0.97 (0.88–1.07)	p = 0.571	0.94 (0.87–1.03)	p = 0.168
Depression scale	baseline		**1.37 (1.12–1.68)**	p = 0.003	**1.29 (1.03–1.61)**	p = 0.025	**1.46 (1.16–1.84)**	p = 0.001	**1.54 (1.26–1.88)**	p<0.001
Peer smoking	baseline		**1.13 (1.03–1.23)**	p = 0.009	**1.12 (1.02–1.24)**	p = 0.019	**1.19 (1.07–1.32)**	p = 0.001	**1.28 (1.18–1.40)**	p<0.001
Likely to have broken rules in school	baseline		**1.18 (1.12–1.24)**	p<0.001	**1.18 (1.12–1.25)**	p<0.001	**1.24 (1.17–1.31)**	p<0.001	**1.37 (1.30–1.44)**	p<0.001
Ever married (at age 26)	baseline		0.93 (0.75–1.15)	p = 0.513	**0.59 (0.47–0.76)**	p<0.001	**0.71 (0.55–0.91)**	p = 0.007	**0.57 (0.46–0.71)**	p<0.001
Has 1+ child (at age 26)	baseline		**1.26 (1.01–1.58)**	p = 0.039	**1.47 (1.15–1.88)**	p = 0.002	**1.61 (1.24–2.08)**	p<0.001	**1.86 (1.49–2.33)**	p<0.001

As tax rates increased, risk of initiation and days of smoking per month decreased in less established users. The effects were most substantial for experimenters (-0.32±0.01 days/month/(ln unit)), never smokers (-0.15±0.02 days/month/(ln unit)) and quitters (-0.02±0.01 days/month/(ln unit)). In contrast, days of use increased for both late escalators (0.83±0.01 days/month/(ln unit) and early established smokers (0.08±0.002 days/month/(ln unit)).

When computing the tax equivalent of a smoke-free law, the largest effect was on quitters, where the smoke-free law was equivalent to a tax well over $1,000, reflecting the fact that the estimated effect of taxes after quitting was so small. The next largest comparative effect was among never smokers, where a smoke-free law was equivalent to a $11.06 tax. The tax-equivalent effects of smoke-laws were more modest among late escalators ($0.85) and early established smokers ($0.52). The equivalent tax effect of smoke-free laws on experimenters was much smaller ($0.08).

Regarding the effects of the sociodemographic risk factors on the probability of membership in each trajectory, compared to never smokers, experimenters were more likely to be younger in 1997; to report being male; having ever used alcohol; having ever used cannabis; higher levels of depression, peer smoking, and a history of rule breaking; and having at least one child (Table 1). Relative to never smokers, late escalators were more likely to report being male; ever using alcohol; ever using cannabis; being younger; higher levels of depression, peer smoking, and a history of rule breaking; and having at least one child. They were less likely to be Hispanic, employed and in school, or have ever been married. Compared to never smokers, quitters were more likely to report being male; being employed and not in school or neither in school nor employed; living in a non-two parent family; ever using alcohol; ever using cannabis; higher levels of depression, peer smoking, and rule breaking; and having at least one child. Quitters were less likely to be non-Hispanic Black or Hispanic, or to have ever been married. Relative to never smokers, early established smokers were more likely to report being male; being employed but not in school or neither in school nor employed; living in a non-two parent family; ever using alcohol; ever using cannabis; higher levels of depression, peer smoking, and rule breaking; and having at least one child. Early established smokers were less likely to be non-Hispanic Black or Hispanic, or to have ever married.

Once respondents had been classified into different groups based on the trajectory analysis, we reviewed the characteristics of the respondents assigned to each trajectory to identify between-trajectory differences (Table 2). We reviewed the characteristics of members of each trajectory; dichotomous variables are reported as percentages, and ordinal variables are reported with means and confidence intervals. Participants identifying as males were more likely to have established smoking habits at a younger age; while less than half of never smokers, experimenters, and late escalators were male, 59% of quitters and 72% of early established smokers were male. While 31% of never smokers reported ever drinking alcohol at baseline, 39% of late escalators, 48% of experimenters, 59% of quitters, and 66% of early established smokers reported ever drinking alcohol. Similarly, 8% of never smokers reported cannabis use at baseline relative to 25% of late escalators, 20% of experimenters, 33% of quitters, and 42% of early established smokers. For the depression, peer smoking, and rule breaking scales, the differences between never smokers and early established smokers ranged between 0.10 and 1.85 on a 5-point scale.

**Table 2:** Individual level characteristics by smoking trajectory.

	Never smokers	Experimenters	Late escalators	Quitters	Early established
	50.8% (n = 2128)	12.5% (n = 527)	9.8% (n = 412)	9.4% (n = 391)	17.5% (n = 734)
	%/mean (95% CI)	%/mean (95% CI)	%/mean (95% CI)	%/mean (95% CI)	%/mean (95% CI)
Gender = male	0.43 (0.41–0.46)	0.52 (0.48–0.56)	0.58 (0.53–0.63)	0.55 (0.50–0.60)	0.50 (0.47–0.54)
Race/ethnicity					
• Non-Hispanic White	0.48 (0.46–0.50)	0.49 (0.44–0.53)	0.46 (0.42–0.51)	0.59 (0.54–0.64)	0.73 (0.69–0.76)
• Non-Hispanic Black	0.28 (0.26–0.30)	0.22 (0.18–0.25)	0.31 (0.26–0.35)	0.23 (0.19–0.27)	0.16 (0.13–0.18)
• Hispanic	0.21 (0.19–0.23)	0.27 (0.24–0.31)	0.18 (0.15–0.22)	0.16 (0.13–0.20)	0.09 (0.07–0.11)
• Non-Hispanic mixed/other	0.03 (0.02–0.04)	0.02 (0.01–0.03)	0.05 (0.03–0.07)	0.02 (0.00–0.03)	0.03 (0.01–0.04)
Mother’s highest education					
• Less than GED/HS diploma	0.15 (0.13–0.16)	0.17 (0.14–0.20)	0.18 (0.15–0.22)	0.14 (0.11–0.18)	0.14 (0.12–0.17)
• GED/HS graduate	0.52 (0.50–0.54)	0.49 (0.45–0.54)	0.53 (0.49–0.58)	0.56 (0.51–0.61)	0.58 (0.54–0.61)
• AA/BA/BS	0.26 (0.25–0.28)	0.28 (0.24–0.32)	0.23 (0.19–0.27)	0.26 (0.22–0.31)	0.24 (0.21–0.28)
• Graduate/professional degree	0.07 (0.06–0.08)	0.06 (0.04–0.08)	0.05 (0.03–0.08)	0.04 (0.02–0.05)	0.04 (0.02–0.05)
Employment/school enrollment status					
• In school, not employed	0.38 (0.36–0.40)	0.37 (0.32–0.41)	0.42 (0.37–0.47)	0.29 (0.25–0.34)	0.26 (0.23–0.30)
• Employed, not in school	0.03 (0.02–0.04)	0.04 (0.03–0.06)	0.05 (0.03–0.07)	0.07 (0.05–0.10)	0.14 (0.12–0.17)
• Employed and in school	0.55 (0.53–0.57)	0.55 (0.51–0.59)	0.46 (0.42–0.51)	0.57 (0.52–0.61)	0.48 (0.44–0.51)
• Neither in school nor employed	0.03 (0.02–0.04)	0.04 (0.02–0.06)	0.06 (0.04–0.09)	0.07 (0.04–0.09)	0.12 (0.09–0.14)
Non-two parent family	0.43 (0.40–0.45)	0.43 (0.38–0.47)	0.50 (0.45–0.55)	0.54 (0.50–0.59)	0.62 (0.58–0.65)
Ever drank alcohol	0.31 (0.29–0.33)	0.48 (0.44–0.52)	0.39 (0.34–0.44)	0.59 (0.54–0.64)	0.66 (0.63–0.69)
Even used cannabis	0.08 (0.07–0.09)	0.20 (0.16–0.23)	0.15 (0.11–0.18)	0.33 (0.29–0.38)	0.42 (0.39–0.46)
Household income	1.93 (1.89–1.97)	1.95 (1.87–2.03)	1.77 (1.68–1.86)	1.94 (1.85–2.03)	1.85 (1.79–1.92)
Age (in 1997)	13.85 (13.79–13.91)	13.82 (13.70–13.94)	13.50 (13.36–13.63)	14.16 (14.02–14.29)	14.14 (14.04–14.25)
Depression scale	0.90 (0.88–0.92)	0.97 (0.93–1.01)	0.94 (0.89–1.00)	0.99 (0.94–1.04)	1.04 (1.00–1.07)
Peer smoking	1.35 (1.30–1.41)	1.55 (1.44–1.66)	1.41 (1.29–1.53)	1.85 (1.73–1.98)	2.07 (1.98–2.17)
Likely to have broken rules in school	1.53 (1.45–1.61)	2.40 (2.22–2.58)	2.46 (2.25–2.66)	2.80 (2.58–3.01)	3.38 (3.22–3.54)
Ever married (at age 26)	0.54 (0.52–0.56)	0.54 (0.50–0.59)	0.40 (0.36–0.45)	0.51 (0.46–0.56)	0.50 (0.46–0.53)
Has 1+ child (at age 26)	0.43 (0.41–0.45)	0.49 (0.45–0.53)	0.51 (0.46–0.56)	0.54 (0.49–0.59)	0.58 (0.55–0.62)

We compared the means and confidence intervals for all variables in the entire NLSY cohort and those in the subset included in the trajectories analysis to assess potential bias in the sample due to missing data. We found that, among the sociodemographic indicators, the subset of observations included in the trajectories analysis had a larger share of respondents identifying as non-Hispanic White and as being both enrolled in school and employed, while a smaller share identified as Hispanic, reported that they were not living with both biological parents and had a mother with less than a GED/high school diploma.

## Discussion

Overall, as anticipated, we identified significant associations between smoking trajectories, tobacco control policy interventions and known risk factors for progression to established smoking. Our findings were consistent with and expand on results from prior research by adding the time-varying effects of two important tobacco policy interventions, smoke-free laws and taxes [8]. In addition, our results demonstrate the effects of socio-demographic variables on patterns of smoking. Our results suggest that policy has different influences on the patterns of smoking behavior of different types of smokers. Our analysis also demonstrated the stronger effects of smoke-free laws on frequency of smoking than tobacco taxes.

Our findings with respect to risk factors for smoking frequency were generally consistent with previous research [7, 8, 10–17], which suggested that white men were more likely to be daily smokers; smoking is associated with alcohol and drug use, peer smoking, and a history of rule breaking; established smoking is associated with lower socioeconomic status; and depression and anxiety are associated with smoking. A previous trajectory analysis using NLSY97 data that did not include time-varying covariates and relied on latent class growth analysis (LCGA) identified roughly comparable shares of experimenters and quitters, smaller shares of never smokers (34.1% versus 50.8% in this study), and larger shares of late escalators (52.0% versus 9.8%) and early established smokers (39.0% versus 17.5%) [8]. With respect to risk factors, confidence intervals in this updated analysis were narrower, identifying significant associations for additional variables in one or more trajectories, including male, being employed and not in school, ever using cannabis, age, depression/anxiety, peer smoking, rule breaking, and having at least one child. In this analysis, non-Hispanic Black participants were also more likely to be experimenters, and Hispanic participants were less likely to be early established smokers.

Our analysis expands upon the existing literature on tobacco control policies and smoking behavior, which focuses on measures of smoking behavior at specific time points, such as initiation, smoking status, and cessation. This is the first analysis to examine the relationship between tobacco control policies and patterns of smoking behavior over time. Our finding that policies have differential effects on smoking trajectories establishes that smokers are heterogeneous, meaning not all smokers follow the same progression to smoking. It may be necessary to tailor cessation interventions to different types of smokers to increase the efficacy of these approaches.

The results also support the importance of tobacco control policy interventions in modifying smoking behavior across all trajectories of use. Comprehensive smoke-free laws were associated with decreased risk of initiation, decreased use, and reduced likelihood of return to use across four out of five trajectories. The effects were greatest for never smokers and quitters, while still evident among established smokers, whether they began smoking as adolescents or as young adults. The only trajectory that did not reduce its exposure to tobacco as a result of coverage by comprehensive smoke-free laws was experimenters.

Our finding that smoke-free laws were not associated with patterns of use among experimenters is consistent with previous literature that established varying effects of smoke-free laws across different patterns of smoking behavior [26, 70]. Siegel et al found that strong smoke-free restaurant laws were associated with lower odds of transitioning from experimentation to established smoking, but not of transitioning from nonsmoking to experimentation [70]. Song et al found that smoke-free laws had a different relationship with smoking initiation, smoking status, and days smoked [26]. Specifically, Song et al found that smoke-free bar laws were associated with lower odds of being a current smoker and fewer days of smoking but not lower odds of smoking initiation [26]. Our findings are also consistent with the intention of smoke-free laws not to prevent experimentation, but to prevent progression from experimentation to established smoking, in addition to protecting nonsmokers from secondhand smoke exposure [71].

The knowledge that experimenters are more likely to have counterintuitive responses to smoke-free laws has the potential to influence tobacco cessation efforts. When a state or locality improves its smoke-free law coverage, it may wish to supplement these changes with smoking prevention and cessation targeting experimenters to ensure that no group fails to benefit from these policy improvements. The analysis that this one builds upon revealed that, compared to never smokers, experimenters were more likely to be neither in school nor working [8]. This finding suggests that school-based tobacco control efforts are less likely to be effective for experimenters than some other types of smokers. Tobacco control programs targeting these youth should be tailored to their use patterns by promoting complete cessation and elimination of occasional or social smoking. These efforts should be placed in locations likely to be frequented by youth who are neither in school nor employed, such as community centers and athletic courts.

While increased tax rates were associated with reduced risk of initiation among never smokers (i.e., a higher cost makes it less appealing to start smoking), reduced days of smoking among experimenters, and reduced likelihood of return to use among quitters, they were associated with increased days of smoking among early established users and late escalators. The finding that established users increase smoking after tax increases is contrary to the intended effect of tobacco tax increases.

In general, because cigarettes are addictive, the relationship between changes in the price and consumption of cigarettes tends to be different from that of many other goods [72]. In addition, previous research suggests that, when tax increases occur, smokers increasingly engage in price minimization strategies such as coupons, bulk purchasing, and switching to discount brands to maintain their prior levels of use [36–41]. In addition, tobacco manufacturer use price promotions to reduce the post-tax consumer prices of cigarettes to levels below the pre-tax prices [43]. Because these changes result in smokers purchasing cigarettes in larger quantities (e.g., carton instead of pack), they also have the potential to result in increased availability, and therefore, increased use. In addition, the use of price minimization strategies and coverage by tobacco-free policies [73] tend to vary by socioeconomic status (SES) [74], and we found differences in some indicators of SES across classes. Policy interventions such as tobacco minimum floor prices or sudden, large tax increases might circumvent the price-minimization strategies likely to be used by established users and late escalators [40, 42, 44, 60].

Future research could consider the effectiveness of these policies by considering changes in smoking trajectories in years beyond 2011, after the introduction of substantial state level annual tax increases (e.g., the 2013 Minnesota tax increase of $1.75 [75]) and local tobacco minimum floor prices (e.g., $7 in Sonoma County, CA in 2016 [76]). In addition, to ensure that all youth benefit from tax increases, states and localities planning tax increases could supplement these increases with cessation methods targeting early established smokers and late escalators. These were the only two trajectories that were significantly more likely to report having frequently broken rules in school compared to never smokers in a previous analysis [8]. School-based efforts and tobacco educational campaigns targeting youth who self-report higher rates of rule-breaking would be most likely to be effective for these types of smokers. In addition, because late escalators do not become established smokers until late adolescence or early adulthood, these programs should extend their reach beyond youth to include young adults by utilizing not only school-based, but also community- and higher education-based smoking prevention and cessation approaches.

### Limitations

Our study has limitations. Our analysis considered annual changes and does not consider policy changes after 2011 when NLSY97 data collection became biennial because the analytic method could not support missing years of data. Because we did not analyze NLSY97 data beyond 2011, we were unable to assess potential interactions between combustible cigarette use and new products such as e-cigarettes and possible complementary use of other substances such as cannabis, which has been increasingly legalized for medical and recreational use [77]. Research using data from the CDC National Youth Tobacco Survey showed that the advent of e-cigarettes had not affected the rate of decline in youth cigarette use from 2004 through 2014 (the last year studied), but that e-cigarettes were adding to nicotine product use [78]. The market for new tobacco products has continued to change, and caution is warranted in attempting to apply these findings to the current market. Our analysis did not include data on Tobacco 21 (T21) laws due to similar limitations. Although biennial NLSY datasets were available through 2018 at the time of writing, the only strong state T21 law in effect before 2019 was California, and organizations that code the strength of Tobacco 21 laws were unable to supply data on local Tobacco 21 laws for any time period. In addition, the switch to a biennial analysis would increase the share of missing data. Our analysis did not include data on state tobacco control funding, for at least two reasons: first, there are multiple differences between states relating to population and focus and quality of programs that make it unclear how to normalize a measure; second, there is likely collinearity between program funding and enactment of smoke-free laws and tax increases, given that stimulating such policy change is often (although not always) among the goals of state tobacco control programs.

We used only a subset of the entire sample due in part to missing geographic identifiers in the underlying data and in part due to incomplete risk factor data (which may reflect social desirability bias); it is unclear whether or how observations excluded due to missing geocodes or incomplete reporting might affect estimates. We relied on listwise deletion as a strategy to handle missing data given that this method is linked to loss of statistical power rather than to biased estimates [79–83]. The fact that we identified statistically significant determinants of the trajectories suggests that this loss in power did not compromise the overall analysis. Another consequence of missing data is that we were unable to include a variable indicating ever use of cocaine/hard drugs, which dropped out of the analysis entirely (in the previous study using NLSY97 data it dropped out of only one trajectory, late escalators [8]).

Another limitation of the analysis is the composition of the sample. A large proportion of the sample was non-Hispanic white and both enrolled in school and employed; a small proportion was Hispanic, not living with both biological parents, and had a mother with less than a GED/high school diploma. As a result, the analysis may not have identified some associations among respondents with underrepresented characteristics.

Future research should consider questions left unanswered by this analysis, including further analysis of the identified increase in smoking days among experimenters under comprehensive smoke-free laws, and among early established and late escalators under higher excise taxes.

### Conclusions

Our findings could help policymakers more effectively target different types of smokers or never smokers with new tobacco control interventions that account for the different trajectories of smoking behavior. Our results suggest that comprehensive smoke-free laws are effective for most smokers but could be supplemented with school- and community-based cessation efforts. Our results also suggest that further interventions are needed to increase the efficacy of tax increases for early established smokers and late escalators. Methods to increase tobacco price increases beyond tax increases, such as minimum floor prices or banning coupons and price promotions, may be more effective deterrents for established smokers and late escalators. These efforts could also be supplemented by school-based interventions that target risk-taking teens and community-based programs targeting youth and young adults.

These findings also provide preliminary data that may guide regulation of new tobacco products, as it is likely that the same types of factors that influence combustible cigarette use are relevant to new products. The implications of this work include the expectation that a combined approach that includes comprehensive smoke-free ordinances, tax increases, and minimum floor prices may be most effective in reducing tobacco consumption across all trajectories of use, throughout adolescence, and into adulthood.
